# The Association Between Physical Activity and Epigastric Pain: A Mendelian Randomization and Cross‐Sectional Study

**DOI:** 10.1155/prm/1787346

**Published:** 2026-06-26

**Authors:** Zhongcao Wei, Qian Yang, Yujie Hao, Na Liu, Jinhai Wang, Xin Xing

**Affiliations:** ^1^ Department of Gastroenterology, The Second Affiliated Hospital of Xi’an Jiaotong University, Shaanxi, China, xjtu.edu.cn; ^2^ Shaanxi Key Laboratory of Gastrointestinal Motility Disorders, Shaanxi, China; ^3^ Shaanxi Provincial Clinical Research Center for Gastrointestinal Diseases, Shaanxi, China; ^4^ Digestive Disease Quality Control Center of Shaanxi Province, Shaanxi, China; ^5^ Department of Gastroenterology, Hainan General Hospital (Hainan Affiliated Hospital of Hainan Medical University), Haikou, Hainan Province, China, hainmc.edu.cn; ^6^ Health Management Department, The Second Affiliated Hospital of Xi’an Jiaotong University, Shaanxi, China, xjtu.edu.cn

**Keywords:** association, cross-sectional study, epigastric pain, Mendelian randomization, physical activity

## Abstract

**Background:**

The relationship between physical activity (PA) and epigastric pain is not well established. This study uniquely combined Mendelian randomization (MR) for causal inference with a cross‐sectional analysis for epidemiological validation.

**Methods:**

We performed an MR analysis using inverse variance weighting (IVW) as the primary method. For the cross‐sectional study, we analyzed data from the National Health and Nutrition Examination Survey (NHANES), categorizing PA (leisure‐time, occupational, and transportation) according to U.S. guidelines.

**Results:**

Genetically predicted walking for pleasure was inversely associated with epigastric pain (IVW OR = 0.966, 95% CI: 0.948–0.985, *P* = 3.912 × 10^−4^). A cross‐sectional analysis revealed contrasting domain‐specific effects: occupation‐related PA meeting guidelines (≥ 150 min/week) increased the risk of epigastric pain (OR: 1.271, 95% CI: 1.119–1.443, *P* = 0.0002), while sufficient leisure‐time PA (≥ 300 minutes/week) was protective (OR: 0.822, 95% CI: 0.693–0.976, *P* = 0.0253). Transportation‐related and total PA showed no significant associations in fully adjusted models.

**Conclusions:**

The integration of genetic and observational evidence demonstrates that the effect of PA on epigastric pain is context‐dependent. Leisure‐time PA is protective, whereas occupation‐related PA may increase risk. These findings highlight the context‐dependent role of PA in gastrointestinal health.

**Highlights**
• Mendelian randomization analysis reveals a significant inverse association between leisure‐time walking and epigastric pain risk.
• Participants achieving ≥ 300 min/week of leisure‐time physical activity exhibited 17.8% lower odds of epigastric pain compared to inactive individuals.
• Guideline‐recommended occupational activity (≥ 150 min/week) showed a significant positive association with epigastric pain risk.
• A dose‐dependent positive relationship was observed between occupational physical activity and epigastric pain across all exposure levels.
• Neither transportation‐related nor total physical activity demonstrated significant associations with epigastric pain in fully adjusted models.

## 1. Introduction

Epigastric pain constitutes a global clinical challenge with significant public health implications, representing one of the most prevalent gastrointestinal complaints in both primary care and emergency settings [1, 2]. It is associated with a range of conditions, including gastroesophageal reflux disease (GERD), peptic ulcer disease, functional dyspepsia [3–5], and, in some cases, malignant processes such as gastric or pancreatic cancers [6, 7]. Despite its high prevalence and considerable healthcare burden, current therapeutic approaches often provide incomplete relief, highlighting the need for better preventive strategies and early risk factor modification.

This unmet clinical need underscores the importance of identifying and modifying risk factors early. Among various lifestyle factors, physical activity (PA) is known to exert beneficial effects on multiple body systems and has been shown to improve symptoms in gastrointestinal conditions [8, 9]. Growing evidence suggests that exercise may also alleviate symptoms of irritable bowel syndrome (IBS) [10–12]. A systematic review of 14 randomized controlled trials (RCTs) evaluated the therapeutic potential of exercise in IBS patients, indicating that PA could serve as an effective intervention [13]. However, the potential role of PA in influencing epigastric pain remains insufficiently studied, with limited evidence regarding its association—whether causal or correlative—with symptom occurrence.

The National Health and Nutrition Examination Survey (NHANES) serves as a comprehensive, population‐based database that systematically collects nationally representative health and nutritional data from the U.S. population [14, 15]. Current PA recommendations, as outlined in the 2018 Physical Activity Guidelines for Americans, provide evidence‐based thresholds for exercise duration and intensity required to achieve meaningful health outcomes. These guidelines specify that optimal health benefits can be attained through weekly participation in either: (1) 150–300 minutes of moderate‐intensity PA, (2) 75–150 minutes of vigorous‐intensity exercise, or (3) an equivalent combination of both activity intensities [16].

To address this gap, we employed a dual‐methodological approach combining Mendelian randomization (MR) with a population‐based epidemiological analysis. Using MR, we aimed to elucidate potential causal genetic links between PA and epigastric pain, while analysis of nationally representative U.S. data from NHANES allowed us to quantify exposure–response relationships across activity types and intensities. We hypothesize that higher levels of PA are associated with a reduced risk of epigastric pain and that this relationship follows a dose–response pattern observable across both genetic and behavioral levels.

### 1.1. MR Study

#### 1.1.1. Study Design

Our instrumental variable analysis was conducted based on three core assumptions of MR: (1) the selected genetic variants must demonstrate statistically significant associations with PA levels; (2) these genetic instruments should remain independent of any known or unknown confounding factors that might influence the outcome; and (3) the genetic variants must exert their effect on epigastric pain exclusively through their impact on PA, without alternative biological pathways.

#### 1.1.2. GWAS Summary Data for PA and Epigastric Pain

Summary‐level genetic association data for PA were obtained from the MRC‐IEU Consortium (*n* = 460376), based on self‐reported activity levels over the last 4 weeks using a validated questionnaire [17]. The present GWAS utilized genetic and phenotypic data derived from the UK Biobank—a population‐based prospective cohort comprising > 500000 participants. This resource provides extensive genomic data coupled with detailed health and lifestyle records, facilitating robust investigation of complex trait determinants. Our analysis focused on six questionnaire‐derived PA phenotypes.

The GWAS examined six distinct PA phenotypes: (1) heavy do‐it‐yourself (DIY) activities (e.g., weeding, lawn mowing, carpentry, digging; ID: ukb‐b‐13184); (2) light DIY activities (e.g., pruning, watering the lawn; ID: ukb‐b‐11495); (3) none of the heavy DIY or light DIY (ID: ukb‐b‐15869); (4) strenuous sports (ID: ukb‐b‐7663); (5) walking for pleasure (ID: ukb‐b‐7337); (6) other exercises (e.g., swimming, cycling, keep fit, bowling; ID: ukb‐b‐8764).

Summary‐level genetic association data for epigastric pain were sourced from the MRC‐IEU Consortium (GWAS ID: ukb‐b‐6608), comprising 463,010 participants of European ancestry from the UK Biobank, including 5981 cases and 457,029 controls. The phenotype was defined as “Pain localised to upper abdomen” (PheCode 41202) derived via the PHESANT pipeline (Version R10.1) based on self‐reported abdominal pain. The GWAS was adjusted for age, sex, 40 genetic principal components, and assessment center. Participants with non‐European ancestry, consent withdrawal, or sex mismatch were excluded. Demographic characteristics of the GWAS population included a mean age of 56.5 years (SD: 8.1), with 54% female participants. All participants included in the MR analysis were of European ancestry (Supporting table 1).

#### 1.1.3. Selection of Genetic Instruments

To create the genetic instrument for PA, single‐nucleotide polymorphisms (SNPs) at the genome‐wide significance level (*P* ≤ 5 × 10^−8^) were obtained, and the linkage disequilibrium (LD) *r*2 < 0.001 with their neighboring variants, in a window of 10,000 kb, and excluded palindromic ones. The genetic instruments need to meet the required threshold of *F* > 10 to ensure the powerful validity, and the *F*‐statistic was calculated using the formula *F* = (β/SE)^2^ [18].

#### 1.1.4. MR Analyses

Inverse variance weighted (IVW) analysis was used as the principal method of analysis. Complementary methods, including MR‐Egger regression, weighted median, weighted mode, and simple mode, were also employed. We prespecified walking for pleasure as the primary exposure due to its biological relevance to leisure‐time activity and prior health associations. The other five activity types were treated as secondary exposures. A Bonferroni‐corrected threshold of *P* < 0.0083 (0.05/6) was applied to the six exposures. A positive result was primarily determined by the significance of IVW (*P* < 0.0083), provided the direction of effect estimates was consistent across methods [19, 20]. In cases of directional inconsistency among methods, a more stringent threshold (*P* ≤ 1 × 10^−8^) was applied to instrumental variables [20, 21]. Details regarding heterogeneity tests, pleiotropy evaluation, outlier detection, and sensitivity analyses are described in the Supporting Methods [22]. All the analyses were performed using *R* 4.2.2.

### 1.2. Patients and Methods

#### 1.2.1. Study Design

This cross‐sectional study used data from the NHANES database over 2017–2020 years (only these 4 years contain information about epigastric pain). The initial sample consisted of 15,560 participants. Participants who met the inclusion criteria and exclusion criteria were included in the study (Supporting table 2). The goal was to analyze the association between PA and epigastric pain.

#### 1.2.2. Data Collection

We collected the basic demographic data (age, sex, BMI, race, country of birth, education, marital status, drinking, current smoking, income (ratio of family income to poverty), hypertension, total cholesterol, glycohemoglobin, diabetes), PA information (vigorous work activity, moderate work activity, walk or bicycle, vigorous recreational activities, moderate recreational activities), and information about epigastric pain. The outcome variable was epigastric pain.

#### 1.2.3. PA

Total PA was quantified by combining three domains: leisure‐time PA, occupation‐related PA, and transportation‐related PA. To standardize the measurement across different intensities, we applied a metabolic equivalent (MET) conversion factor, wherein vigorous‐intensity PA minutes were weighted by a factor of 2 before being combined with moderate‐intensity PA minutes [23]. Following the 2018 Physical Activity Guidelines for Americans, participants were classified based on their weekly PA engagement: Those achieving either ≥ 150 minutes of moderate‐intensity PA, 75 minutes of vigorous‐intensity PA, or an equivalent combination were categorized as meeting the guidelines [24].

### 1.3. Statistical Analyses

Statistical analyses were performed by EmpowerStats and SPSS 20.0 (IBM Corp., Armonk, New York, USA). Categorical variables were reported as counts and percentages and analyzed by chi‐square tests or Fisher’s exact test. Continuous variables were assessed for normality using the Shapiro–Wilk test, supported by visual inspection of Q–Q plots and histograms. Data were reported as the mean ± SD and analyzed by a *t*‐test or Kruskal–Wallis test. All variables were evaluated with univariate analysis. Multivariate logistic modeling was used to evaluate the association of PA and epigastric pain, Model 1 was unadjusted, Model 2 adjusted for age and sex. Model 3 adjusted for age, sex, BMI, race, country of birth, education, marital status, drinking, current smoking, income, hypertension, total cholesterol, glycohemoglobin, and diabetes. Data were presented with OR and 95% confidence intervals (CIs). *P* < 0.05 was considered statistically significant.

## 2. Results

### 2.1. MR Study

#### 2.1.1. Study Design

The analysis included 5981 epigastric pain cases and 457029 controls. Following SNP selection criteria, we identified the following instrumental variables: heavy DIY activity: 17 SNPs (*F*‐statistic range: 30.6–51.7); light DIY activity: 12 SNPs (*F*‐statistic range: 29.8–84.9); none of the heavy DIY or light DIY activity: 5 SNPs (*F*‐statistic range: 31.3–32.9); strenuous sports: 6 SNPs (*F*‐statistic range: 34.5–49.2); walking for pleasure: 20 SNPs (*F*‐statistic range: 30.5–38.8); other exercises (e.g., swimming, cycling, keep fit, bowling): 15 SNPs (*F*‐statistic range: 30.3–61.1). The characteristics of instrumental variables for PA exposures are shown in Supporting Table 3. All selected SNPs exhibited strong associations with epigastric pain (*F*‐statistics > 10), indicating robust instrument strength. Data sources and variant details are provided in Supporting Table 1.

#### 2.1.2. Causal Effect of PA on Epigastric Pain

Among all types of PA assessed, only walking for pleasure showed a significant causal effect on epigastric pain. Using the IVW method, walking for pleasure was associated with a reduced risk of epigastric pain (OR = 0.966; 95% CI, 0.948–0.985; *P* = 3.912 × 10^−4^). The weighted median method also supported this suggestive association (OR = 0.963; 95% CI, 0.938–0.988; *P* = 3.648 × 10^−3^). Sensitivity analyses, including MR‐Egger regression and MR‐PRESSO, detected no significant horizontal pleiotropy or outliers (MR‐Egger intercept *P* = 0.909; MR‐PRESSO *P* = 0.825). Leave‐one‐out analysis confirmed that the result was not driven by any single genetic variant (Table 1). Post hoc statistical power was estimated using the mRnd online tool. Given the variance explained by our genetic instruments (*R* ^2^ = 0.15% for walking for pleasure), the observed effect size (OR = 0.966), and a Bonferroni‐corrected *α* of 0.0083, the estimated power was approximately 1%. This low power underscores that our significant finding is unlikely to be a chance event and suggests possible attenuation of the true effect due to weak instrument bias. Future studies with stronger genetic instruments or larger samples are needed for more precise estimation.

**TABLE 1:** MR results for the relationship between physical activity and epigastric pain.

Exposure	Method	OR (95% CI)	*P* value	SNPs	Heterogeneity test (*p* value)	Pleiotropy test (*p* value)
Heavy DIY	IVW (fixed)	0.990 (0.969–1.010)	0.326	17	0.241	
	MR‐Egger	0.842 (0.720–0.985)	0.048	17	0.428	0.060
	Weighted median	0.991 (0.965–1.018)	0.499	17		
	Simple mode	0.986 (0.937–1.037)	0.593	17		
	Weighted mode	0.987 (0.935–1.042)	0.643	17		
Light DIY	IVW (random)	0.976 (0.950–1.003)	0.081	12	0.044	
	MR‐Egger	0.935 (0.823–1.062)	0.327	12	0.037	0.514
	Weighted median	0.982 (0.954–1.011)	0.223	12		
	Simple mode	0.997 (0.946–1.051)	0.912	12		
	Weighted mode	0.986 (0.943–1.030)	0.533	12		
None of the heavy DIY or light DIY	IVW (fixed)	0.989 (0.920–1.063)	0.761	5	0.458	
	MR‐Egger	1.420 (0.616–3.278)	0.471	5	0.406	0.457
	Weighted median	1.001 (0.910–1.102)	0.977	5		
	Simple mode	1.027 (0.900–1.171)	0.713	5		
	Weighted mode	1.003 (0.855–1.176)	0.768	5		
Strenuous sports	IVW (fixed)	0.967 (0.922–1.015)	0.177	6	0.887	
	MR‐Egger	0.999 (0.802–1.243)	0.992	6	0.804	0.783
	Weighted median	0.978 (0.924–1.036)	0.451	6		
	Simple mode	0.986 (0.906–1.073)	0.754	6		
	Weighted mode	0.985 (0.910–1.067)	0.731	6		
Walking for pleasure	IVW (fixed)	0.966 (0.948–0.985)	3.912e^−4^	20	0.835	
	MR‐Egger	0.952 (0.741–1.224)	0.707	20	0.789	0.909
	Weighted median	0.963 (0.938–0.988)	3.648e^−3^	20		
	Simple mode	0.953 (0.910–0.998)	0.057	20		
	Weighted mode	0.957 (0.909–1.006)	0.102	20		
Other exercises (e.g., swimming, cycling, keep fit, bowling)	IVW (fixed)	0.983 (0.963–1.005)	0.131	15	0.258	
	MR‐Egger	0.963 (0.816–1.135)	0.659	15	0.203	0.798
	Weighted median	0.977 (0.951–1.004)	0.095	15		
	Simple mode	0.969 (0.921–1.020)	0.255	15		
	Weighted mode	0.969 (0.924–1.016)	0.219	15		

*Note:* If there was no heterogeneity, a fixed‐effects model was used, and if heterogeneity was found, a random‐effects model was used.

Abbreviations: IVW = inverse variance weighted, SNPs = single‐nucleotide polymorphisms.

In contrast, no significant causal associations were observed for heavy DIY, light DIY, none of the heavy DIY or light DIY, strenuous sports, or other exercises (e.g., swimming, cycling, keep fit, bowling) (Table 1). Detailed results for these associations are provided in Supporting Results.

### 2.2. Cross‐Sectional Study

#### 2.2.1. Study Population and Baseline Characteristics

**FIGURE 1:**
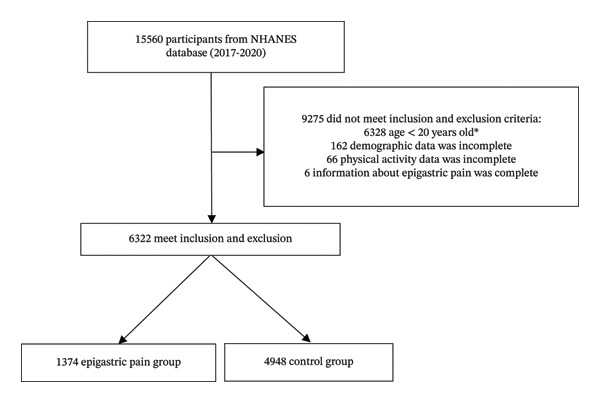
Participant selection flow diagram for the cross‐sectional analysis of physical activity and epigastric pain. The figure illustrates the process of selecting eligible participants from the NHANES 2017–2020 cycle. From an initial pool of 15560 individuals, 9275 were excluded for not meeting the study criteria. Exclusions were primarily due to age below 20 years (*n* = 6328), followed by incomplete demographic data (*n* = 162), missing physical activity data (*n* = 66), and unavailable epigastric pain status (*n* = 6). The final analytical sample consisted of 6322 adults, categorized into an epigastric pain group (*n* = 1374) and a control group (*n* = 4948). (∗: The NHANE database counts age ≥ 20 years as an adult).

A total of 15560 participants from the NHANES 2017–2020 database about epigastric pain was the study’s data source. And 6322 participants were included in the final analysis, comprising 1374 individuals with epigastric pain and 4948 controls (Figure 1). The baseline characteristics of the study population are summarized in Table 2. Univariate analysis revealed significant differences between the epigastric pain and control groups in several demographic and metabolic factors, including sex, BMI, race, country of birth, education level, marital status, income, current smoking, hypertension, diabetes, and occupation‐related PA ≥ 150 minutes/week and leisure‐time PA ≥ 150 minutes/week (all *P* < 0.05).

**TABLE 2:** Baseline characteristics of all participants and univariate analyses for epigastric pain.

Characteristics	Epigastric pain group (*n* = 1374)	Control group (*n* = 4948)	*P* value
Age, years	50.7 ± 17.2	50.8 ± 17.4	0.801
Sex (%)			< 0.001
Male	612 (44.5)	2478 (50.1)	
Female	762 (55.5)	2470 (49.9)	
BMI	30.6 ± 7.7	30.0 ± 7.5	0.002
Race (%)			< 0.001
Mexican American	165 (12.0)	561 (11.3)	
Other Hispanic	157 (11.4)	466 (9.4)	
Non‐Hispanic White	570 (41.5)	1830 (37.0)	
Non‐Hispanic Black	305 (22.2)	1255 (25.4)	
Other Race	177 (12.9)	836 (16.9)	
Country of birth, (%)			< 0.001
50 US states or Washington	1065 (77.5)	3588 (72.5)	
Others	309 (22.5)	1360 (27.5)	
Education (%)			< 0.001
< 9th grade	99 (7.2)	280 (5.7)	
9–11 grade	173 (12.6)	491 (9.9)	
High school graduate	356 (25.9)	1163 (23.5)	
Some college or AA degree	490 (35.7)	1662 (33.6)	
College graduate or above	256 (18.6)	1352 (27.3)	
Marital status (%)			0.043
Married	772 (56.2)	2964 (59.9)	
Widowed/divorced	331 (24.1)	1075 (21.7)	
Never married	271 (19.7)	909 (18.4)	
Income (ratio)	2.3 ± 1.6	2.7 ± 1.6	< 0.001
Drinking (%)	1274 (92.7)	4520 (91.4)	0.104
Current smoking (%)	395 (26.1)	1018 (20.6)	< 0.001
Hypertension (%)	612 (44.5)	1804 (36.5)	< 0.001
Total cholesterol (mg/dL)	185.8 ± 43.0	186.0 ± 40.9	0.603
Glycohemoglobin	5.9 ± 1.2	5.8 ± 1.1	0.405
Diabetes (%)	262 (19.1)	704 (14.2)	< 0.001
Occupation‐related physical activity (minutes/week)			< 0.001
< 150	719 (52.3)	2924 (59.1)	
≥ 150	655 (47.7)	2024 (40.9)	
Transportation‐related physical activity (minutes/week)			0.950
< 150	1221 (88.9)	4400 (88.9)	
≥ 150	153 (11.1)	548 (11.1)	
Leisure‐time physical activity (minutes/week)			< 0.001
< 150	979 (71.3)	3198 (64.6)	
≥ 150	395 (28.7)	1750 (35.4)	
Total physical activity (minutes/week)			0.740
< 150	489 (35.6)	1785 (36.1)	
≥ 150	885 (64.4)	3163 (63.9)	

*Note:* Values are expressed as the mean ± standard deviation or *n* (%).

#### 2.2.2. Association Between PA and Epigastric Pain

Multivariable logistic regression analyses were performed to examine the association between meeting PA guidelines (≥ 150 minutes/week) and epigastric pain (Table 3). After full adjustment for demographic and metabolic confounders, occupation‐related PA meeting the guidelines was significantly associated with an increased odds of epigastric pain (OR: 1.271; 95% CI, 1.119–1.443; *P* = 0.0002). In contrast, leisure‐time PA meeting the guidelines was not significantly associated with epigastric pain in the fully adjusted model (OR: 0.885; 95% CI, 0.768–1.018; *P* = 0.088).

**TABLE 3:** Multivariable OR for epigastric pain based on the meeting physical activity guideline (≥ 150 min/wk).

	Model 1 OR (95% CI), *P* value	Model 2 OR (95% CI), *P* value	Model 3 OR (95% CI), *P* value
Occupation‐related physical activity (minutes/week)			
< 150	1.0	1.0	1.0
≥ 150	1.316 (1.167, 1.484) *P* < 0.0001	1.368 (1.210, 1.547) *P* < 0.0001	1.271 (1.119, 1.443) *P* = 0.0002
Transportation‐related physical activity (minutes/week)			
< 150	1.0	1.0	1.0
≥ 150	1.006 (0.832, 1.217) *P* = 0.9499	1.037 (0.856, 1.256) *P* = 0.7115	0.988 (0.811, 1.203) *P* = 0.9030
Leisure‐time physical activity (minutes/week)			
< 150	1.0	1.0	1.0
≥ 150	0.737 (0.647, 0.840) *P* < 0.0001	0.744 (0.651, 0.850) *P* < 0.0001	0.885 (0.768, 1.018) *P* = 0.0876
Total physical activity (minutes/week)			
< 150	1.0	1.0	1.0
≥ 150	1.021 (0.902, 1.157) *P* = 0.7400	1.059 (0.930, 1.205) *P* = 0.3884	1.123 (0.983, 1.283) *P* = 0.0880

*Note:* Model 1 unadjusted; Model 2: adjusted for age and sex; Model 3: adjusted for age, sex, BMI, race, country of birth, education, marital status, income, drinking, current smoking, income, hypertension, total cholesterol, glycohemoglobin, and diabetes.

A dose–response analysis further categorized PA into four groups (0, 1–149, 150–299, and ≥ 300 minutes/week) to assess the dose–response relationship between various types of PA and epigastric pain (Table 4). Higher levels of leisure‐time PA (≥ 300 minutes/week) were associated with significantly lower odds of epigastric pain (OR: 0.822; 95% CI, 0.693–0.976; *P* = 0.025). Conversely, all levels of occupation‐related PA showed significantly increased odds, with the strongest association observed at 1–149 minutes/week (OR: 1.539; 95% CI, 1.220–1.943; *P* = 0.0003). No significant associations were found for transportation‐related PA or total PA. These findings suggest that while higher levels of leisure‐time PA (≥ 300 minutes/week) may reduce the risk of epigastric pain, higher levels of occupation‐related PA may increase the risk, highlighting the importance of the type and context of PA in relation to epigastric pain.

**TABLE 4:** Multivariable OR for epigastric pain based on the amount of physical activity.

	Model 1 OR (95% CI), *P* value	Model 2 OR (95% CI), *P* value	Model 3 OR (95% CI), *P* value
Occupation‐related physical activity (minutes/week)			
0	1.0	1.0	1.0
1–149	1.497 (1.192, 1.880) *P* = 0.0005	1.512 (1.204, 1.899) *P* = 0.0004	1.539 (1.220, 1.943) *P* = 0.0003
150–299	1.325 (1.004, 1.748) *P* = 0.0465	1.349 (1.022, 1.781) *P* = 0.0343	1.354 (1.019, 1.799) *P* = 0.0366
≥ 300	1.401 (1.231, 1.594) *P* < 0.0001	1.464 (1.282, 1.671) *P* < 0.0001	1.352 (1.179, 1.551) *P* < 0.0001
Transportation‐related physical activity (minutes/week)			
0	1.0	1.0	1.0
1–149	0.854 (0.700, 1.043) *P* = 0.1217	0.862 (0.705, 1.053) *P* = 0.1460	0.867 (0.705, 1.065) *P* = 0.1725
150–299	0.827 (0.621, 1.101) *P* = 0.1931	0.847 (0.635, 1.130) *P* = 0.2587	0.817 (0.609, 1.096) *P* = 0.1769
≥ 300	1.134 (0.888, 1.447) *P* = 0.3138	1.175 (0.918, 1.502) *P* = 0.1998	1.105 (0.858, 1.424) *P* = 0.4375
Leisure‐time physical activity (minutes/week)			
0	1.0	1.0	1.0
1–149	0.883 (0.740, 1.053) *P* = 0.1662	0.874 (0.733, 1.044) *P* = 0.1367	0.997 (0.831, 1.196) *P* = 0.9712
150–299	0.844 (0.690, 1.031) *P* = 0.0963	0.843 (0.689, 1.031) *P* = 0.0955	1.004 (0.816, 1.236) *P* = 0.9694
≥ 300	0.661 (0.565, 0.775) *P* < 0.0001	0.665 (0.565, 0.782) *P* < 0.0001	0.822 (0.693, 0.976) *P* = 0.0253
Total physical activity (minutes/week)			
0	1.0	1.0	1.0
1–149	0.874 (0.704, 1.085) *P* = 0.2232	0.877 (0.706, 1.089) *P* = 0.2338	0.972 (0.779, 1.212) *P* = 0.7989
150–299	0.887 (0.703, 1.120) *P* = 0.3137	0.899 (0.712, 1.136) *P* = 0.3732	1.045 (0.823, 1.328) *P* = 0.7164
≥ 300	0.995 (0.861, 1.150) *P* = 0.9426	1.039 (0.893, 1.208) *P* = 0.6214	1.126 (0.964, 1.315) *P* = 0.1339

*Note:* Model 1 unadjusted; Model 2: adjusted for age and sex; Model 3: adjusted for age, sex, BMI, race, country of birth, education, marital status, income, drinking, current smoking, income, hypertension, total cholesterol, glycohemoglobin, and diabetes.

## 3. Discussion

Our MR analysis revealed a significant inverse causal relationship between walking for pleasure—a type of leisure‐time PA—and epigastric pain. This genetic evidence is further supported by cross‐sectional observations, in which engaging in ≥ 300 minutes per week of leisure‐time PA was associated with a 17.8% reduction in the odds of epigastric pain (OR: 0.822; 95% CI, 0.693–0.976; *P* = 0.025) after full adjustment for confounders. In contrast, all levels of occupation‐related PA were consistently associated with an elevated risk of epigastric pain. These results highlight not only the protective role of leisure‐time PA but also the potential adverse effects of occupational activity on abdominal health.

Our findings align with previous research demonstrating the protective effects of leisure‐time PA on various health outcomes, including nonalcoholic fatty liver disease (NAFLD) [25, 26] and cardiovascular diseases [27]. The dose‐dependent relationship observed in our study suggests that exceeding the recommended PA guidelines may provide additional benefits in reducing the risk of epigastric pain. This protective effect may be mediated by reductions in systemic inflammation, improvements in metabolic health, and enhanced gastrointestinal motility associated with regular PA [28–30].

Conversely, the detrimental association observed with occupational PA may be attributed to several context‐specific mechanisms. Unlike leisure‐time PA, occupational activity often involves sustained physical strain, repetitive motion, prolonged sedentary postures, or heavy lifting, which can induce intra‐abdominal pressure changes and visceral stress [31, 32]. Additionally, occupational PA is frequently accompanied by psychosocial stressors such as low job control, time pressure, and limited recovery periods, which may amplify physiological stress responses and promote gastrointestinal symptoms. Transportation‐related PA and total PA did not show significant associations with epigastric pain. This lack of association may be due to the relatively lower intensity or shorter duration of transportation‐related PA compared to leisure‐time or occupation‐related PA. The absence of association with transportation‐related PA further underscores that not all PA is equivalent—context, voluntariness, and nature of movement appear to critically influence gastrointestinal outcomes.

These findings carry meaningful implications for public health messaging and clinical guidance. While leisure‐time PA should continue to be encouraged, occupational settings may require tailored interventions—such as ergonomic adjustments, structured breaks, and stress management programs—to mitigate the gastrointestinal risks associated with work‐related physical exertion. Future research should aim to clarify the physiological and psychosocial pathways through which different types of PA influence abdominal health, enabling more precise and effective preventive strategies.

This study has several strengths, including the combination of MR and a large, nationally representative cross‐sectional sample. However, some limitations should be acknowledged. First, the cross‐sectional nature of the NHANES data prevents the establishment of causal inference between PA and epigastric pain. Second, the MR analysis was conducted using genetic data from individuals of European ancestry, which may limit the generalizability of the causal estimates to other ancestral populations. Third, our MR analyses were statistically underpowered (estimated power 1.0%) to detect the observed small effect sizes, reflecting the limited variance explained by the genetic instruments for complex behavioral traits like PA. The fact that walking for pleasure nevertheless showed a significant association (*P* = 3.912 × 10^−4^) suggests that the true causal effect may be larger than our point estimate indicates (possibly attenuated by weak instrument bias). These findings should be interpreted with appropriate caution, and future studies with stronger genetic instruments or larger samples are needed for more precise estimation. Fourth, PA levels were self‐reported, which is susceptible to recall and social desirability bias. Fifth, we were unable to account for dietary habits or other lifestyle factors that may confound the association between PA and epigastric pain. Future longitudinal studies incorporating objective PA measurements, detailed dietary assessment, and diverse populations are warranted to confirm these findings and elucidate the underlying mechanisms. Finally, our primary outcome (epigastric pain) is a broad symptom that may arise from multiple etiologies (e.g., GERD, peptic ulcer, gastritis, functional dyspepsia). The mechanisms through which PA influences these conditions may differ. Therefore, our effect estimate represents an average association across heterogeneous pathways, which may dilute specific causal effects. Future studies with more precise phenotyping are needed.

In conclusion, this study reveals contrasting effects of PA on epigastric pain risk: Leisure‐time PA demonstrates a dose‐dependent protective effect, while occupation‐related PA may increase risk. These findings highlight the importance of distinguishing between activity contexts in health guidelines and clinical practice. Future research should investigate the underlying mechanisms, including stress physiology and activity intensity, and develop targeted interventions to optimize PA benefits while mitigating occupational risks. This work challenges the assumption of uniform health effects across all PA domains, advancing our understanding of context‐dependent PA outcomes.

**Nomenclature**
CIs: Confidence intervals
GERD: Gastroesophageal reflux disease
IBS: Irritable bowel syndrome
IVW: Inverse variance weighting
LD: Linkage disequilibrium
MR: Mendelian randomization
NAFLD: Nonalcoholic fatty liver disease
NHANES: National Health and Nutrition Examination Survey
PA: Physical activity
SNPs: Single‐nucleotide polymorphisms

## Author Contributions

Zhongcao Wei, Jinhai Wang, Xin Xing, and Na Liu designed the study. Xin Xing and Qian Yang collected the data. Zhongcao Wei and Yujie Hao performed statistical analysis. Zhongcao Wei, Xin Xing, Jinhai Wang, and Na Liu wrote the manuscript.

## Funding

This work was supported by the Key Project of Shaanxi Province (Grant Nos. 2018ZDXM‐SF‐046, 2020SF‐159).

## Disclosure

All authors finally approved the manuscript.

## Ethics Statement

The National Health and Nutrition Examination Survey was approved by the NCHS Ethics Review Board (ERB). This study was conducted in accordance with the 1964 Declaration of Helsinki and its later amendments.

## Consent

All participants provided written informed consent.

## Conflicts of Interest

The authors declare no conflicts of interest.

## Supporting Information

Additional supporting information can be found online in the Supporting Information section.

## Supporting information


**Supporting Information** Supporting Methods: Description of genetic harmonization procedures, handling of palindromic SNPs, heterogeneity assessment using Cochran’s *Q* test, model selection (fixed vs. random effects), outlier detection using MR‐PRESSO, pleiotropy assessment via MR‐Egger intercept, and leave‐one‐out sensitivity analysis. Supporting Results: Detailed MR results for each physical activity exposure (heavy DIY, light DIY, none of heavy/light DIY, strenuous sports, walking for pleasure, and other exercises) on epigastric pain, including heterogeneity statistics, causal estimates (OR, 95% CI, *p* values), pleiotropy tests, outlier detection, and leave‐one‐out findings. Supporting Table 1: Data sources and GWAS identifiers for physical activity exposures and epigastric pain outcomes, including sample sizes and SNP counts. Supporting Table 2: Inclusion and exclusion criteria for participant selection in the study population. Supporting Table 3: Characteristics of instrumental variables (SNPs) for each physical activity exposure, including effect allele, beta, effect allele frequency (EAF), standard error, *p* value, and *F*‐statistic (subdivided into panels A–*F* for each activity type). Supporting Figure 1: Forest plots showing the causal effects of individual and combined SNPs for each physical activity on epigastric pain, with inverse variance weighted (IVW) results. Supporting Figure 2: Scatterplots illustrating the relationship between SNP effects on physical activity and SNP effects on epigastric pain for each exposure. Supporting Figure 3: Leave‐one‐out sensitivity analysis plots for each physical activity exposure, demonstrating the stability of MR results when individual SNPs are sequentially removed.

## Data Availability

The NHANES data used in the cross‐sectional analysis are publicly available from the National Center for Health Statistics website: https://www.cdc.gov/nchs/nhanes/. The GWAS summary statistics for physical activity and epigastric pain were obtained from the MRC‐IEU OpenGWAS database (https://gwas.mrcieu.ac.uk/). The analysis code used in this study is available upon reasonable request from the corresponding author.
