# Supplementary material for: The Association Between Physical Activity and Epigastric Pain: A Mendelian Randomization and Cross‐Sectional Study
Source: Pain Res Manag. 2026 Jun 26;2026:1787346. doi: 10.1155/prm/1787346 (PMC13309679; doi:10.1155/prm/1787346)
Supplement: Supplementary file 1 — Supporting Information Supporting Methods: Description of genetic harmonization procedures, handling of palindromic SNPs, heterogeneity assessment using Cochran’s Q test, model selection (fixed vs. random effects), outlier detection using MR‐PRESSO, pleiotropy assessment via MR‐Egger intercept, and leave‐one‐out sensitivity analysis. Supporting Results: Detailed MR results for each physical activity exposure (heavy DIY, light DIY, none of heavy/light DIY, strenuous sports, walking for pleasure, and other exercises) on epigastric pain, including heterogeneity statistics, causal estimates (OR, 95% CI, p values), pleiotropy tests, outlier detection, and leave‐one‐out findings. Supporting Table 1: Data sources and GWAS identifiers for physical activity exposures and epigastric pain outcomes, including sample sizes and SNP counts. Supporting Table 2: Inclusion and exclusion criteria for participant selection in the study population. Supporting Table 3: Characteristics of instrumental variables (SNPs) for each physical activity exposure, including effect allele, beta, effect allele frequency (EAF), standard error, p value, and F‐statistic (subdivided into panels A–F for each activity type). Supporting Figure 1: Forest plots showing the causal effects of individual and combined SNPs for each physical activity on epigastric pain, with inverse variance weighted (IVW) results. Supporting Figure 2: Scatterplots illustrating the relationship between SNP effects on physical activity and SNP effects on epigastric pain for each exposure. Supporting Figure 3: Leave‐one‐out sensitivity analysis plots for each physical activity exposure, demonstrating the stability of MR results when individual SNPs are sequentially removed. [file PRM-2026-1787346-s001.docx]

**Supplementary Methods**

Genetic harmonization was performed using TwoSampleMR (v0.5.6). Palindromic SNPs (A/T or C/G) were identified, and allele frequencies (EAF/MAF) were used to infer strand orientation where possible. To avoid ambiguity, palindromic SNPs with MAF > 0.42 were excluded. Remaining SNPs were harmonized using harmonise_data() with default settings. Instrument details are provided in Supplementary Table S1; code is available upon request. The Cocrane’s Q test was used to determine the presence of heterogeneous, and *P* < 0.05 was considered significant. If there was no heterogeneity, a fixed effects model was used, and if heterogeneity was found, a random effects model was used. MR-PRESSO was used to detect the existence of outliers, and the intercept obtained by MR-Egger regression determines whether there was pleiotropy, *P* < 0.05 was considered significant. The leave-one-out analysis was used to evaluate the stability of MR Results.

**Supplementary Results**

**Causal effect from** **Heavy DIY to epigastric pain**

Q values of MR-Egger and IVW showed no significant heterogeneity between Heavy DIY and FD (*P* = 0.428, and 0.241, respectively) and fixed effect model was used. We found that Heavy DIY had no causality on epigastric pain (IVW OR = 0.990; 95% CI, 0.969–1.010; *P* = 0.326) (Table 1). The forest plot of the effects of single and combined SNPs of Heavy DIY on epigastric pain was shown in supplementary figure 1A, and the scatterplot of the effects of SNPs on epigastric pain versus their effects on Heavy DIY was shown in supplementary figure 2A. MR-Egger regression found no evidence of potential pleiotropy (*P* intercept =0.060), and no outlier SNPs were detected by MR-PRESSO method (*P* = 0.237). The leave-one method was used for sensitivity analysis, which showed no effect by a single study (Supplementary figure 3A).

**Causal effect from Light DIY to epigastric pain**

According to Cochran Q-test, the p value of MR-Egger was 0.037 and that of IVW was 0.044, indicating the existence of heterogeneity, and random effect model was used. We found that Light DIY had no causality on FD (IVW OR = 0.976; 95% CI, 0.950–1.003; *P* = 0.081) (Table 1). The forest plot of the effects of single and combined SNPs of light DIY on epigastric pain was shown in supplementary figure 1B, and the scatterplot of the effects of SNPs on epigastric pain versus their effects on light DIY was shown in supplementary figure 2B. MR-Egger regression found no evidence of potential pleiotropy (*P* intercept =0.514), and no outlier SNPs were detected by MR-PRESSO method (*P* = 0.07). The leave-one method was used for sensitivity analysis, which showed no effect by a single study (Supplementary figure 3B).

**Causal effect from** **none of the Heavy DIY** **or Light DIY to epigastric pain**

According to Cochran Q-test, the p value of MR-Egger was 0.406 and that of IVW was 0.458, indicating no significant heterogeneity, and fixed effect model was used. We found that none of the Heavy DIY or Light DIY had no causality on FD (IVW OR = 0.989; 95% CI, 0.920–1.063; *P* = 0.761) (Table 1). The forest plot of the effects of single and combined SNPs of none of the heavy DIY or light DIY on epigastric pain was shown in supplementary figure 1C, and the scatterplot of the effects of SNPs on epigastric pain versus their effects on none of the heavy DIY or light DIY was shown in supplementary figure 2C. MR-Egger regression found no evidence of potential pleiotropy (*P* intercept =0.457), and no outlier SNPs were detected by MR-PRESSO method (*P* = 0.487). The leave-one method was used for sensitivity analysis, which showed no effect by a single study (Supplementary figure 3C).

**Causal effect from strenuous sports to epigastric pain**

In the causality analysis of strenuous sports on epigastric pain, heterogeneity was not found with a Cochran Q-test derived *P* value as 0.804 of MR-Egger and p value as 0.887 of IVW, and fixed effect model was used. The result of IVW indicated that strenuous sports had no causality on epigastric pain (OR = 0.967, 95% CI = 0.922-1.015, *P* = 0.177) (Table 1). The forest plot of the effects of single and combined SNPs of strenuous sports on epigastric pain was shown in supplementary figure 1D, and the scatterplot of the effects of SNPs on epigastric pain versus their effects on strenuous sports was shown in supplementary figure 2D. MR-PRESSO global test (*P* = 0.898) showed that there was no existence of outliers. MR-Egger regression intercept (*P* = 0.783) did not detect potential horizontal pleiotropy. Leave-one-out analysis showed no influence of individual studies (Supplementary figure 3D).

**Causal effect from** **walking for pleasure to epigastric pain**

Q values of MR-Egger and IVW showed no significant heterogeneity between walking for pleasure and epigastric pain (*P* = 0.789, and 0.835, respectively) and fixed effect model was used. The result of IVW indicated that walking for pleasure had causality on epigastric pain (OR = 0.966, 95% CI = 0.948-0.985, *P* = 3.912e^-4^), and the results of the Weighted media also showed a suggestive association (OR = 0.963; 95% CI, 0.938–0.988; *P* = 3.648e^-3^) (Table 1). The forest plot of the effects of single and combined SNPs of walking for pleasure on epigastric pain was shown in supplementary figure 1E, and the scatterplot of the effects of SNPs on epigastric pain versus their effects on walking for pleasure was shown in supplementary figure 2E. And the IVW method had the same OR direction as the other methods. The MR-Egger intercept did not detect the presence of potential horizontal pleiotropy (*P* = 0.909), and no MR-PRESSO outliers were detected (*P* = 0.825). Leave-one-out analysis showed no influence of individual studies (Supplementary figure 3E).

**Causal effect from other exercises (eg: swimming, cycling, keep fit, bowling) to epigastric pain**

According to Cochran Q-test, the p value of MR-Egger was 0.203 and that of IVW was 0.258, indicating no significant heterogeneity, and fixed effect model was used. We found that other exercises (eg: swimming, cycling, keep fit, bowling) had no causality on epigastric pain (IVW OR = 0.983; 95% CI, 0.963–1.005; *P* = 0.131) (Table 1). The forest plot of the effects of single and combined SNPs of other exercises on epigastric pain was shown in supplementary figure 1F, and the scatterplot of the effects of SNPs on epigastric pain versus their effects on other exercises was shown in supplementary figure 2F. MR-Egger regression found no evidence of potential pleiotropy (*P* intercept =0.798), and no outlier SNPs were detected by MR-PRESSO method (*P* = 0.308). The leave-one method was used for sensitivity analysis, which showed no effect by a single study (Supplementary figure 3F).

**Supplementary table 1:** Data sources and IDs.

| GWAS-ID | Phenotype | Year | Population | Sex | Cases | Controls | Sample size | SNPs |
| --- | --- | --- | --- | --- | --- | --- | --- | --- |
| ukb-b-13184 | Heavy DIY | 2018 | European | M&F | 197,006 | 263,370 | 460,376 | 9,851,867 |
| ukb-b-11495 | Light DIY | 2018 | European | M&F | 236,244 | 224,132 | 460,376 | 9,851,867 |
| ukb-b-15869 | None of the Heavy DIY or light DIY | 2018 | European | M&F | 28,040 | 432,336 | 460,376 | 9,851,867 |
| ukb-b-7663 | Strenuous sports | 2018 | European | M&F | 47,468 | 412,908 | 460,376 | 9,851,867 |
| ukb-b-7337 | Walking for pleasure | 2018 | European | M&F | 329,755 | 130,621 | 460,376 | 9,851,867 |
| ukb-b-8764 | Other exercises | 2018 | European | M&F | 222,470 | 237,906 | 460,376 | 9,851,867 |
| ukb-b-6606 | Epigastric pain | 2018 | European | M&F | 5,981 | 457,029 | 463,010 | 9,851,867 |

M&F: Males and Females.

**Supplementary table 2:** Inclusion criteria and Exclusion criteria.

| Criterion | Description |
| --- | --- |
| Inclusion criteria | 1) age 20 years and older;  2) basic demographic data, physical activity information and information about epigastric pain were complete. |
| Exclusion criteria | 1) participants were younger than 20 years old (the NHANE database counts age ≥20 years as an adult);  2) incomplete data of participants. |

**Supplementary Table 3:** Characteristics of instrumental variables for physical activity exposures. **A**

| SNP | Effect allele | Other allele | beta | eaf | se | p | F |
| --- | --- | --- | --- | --- | --- | --- | --- |
| rs10200379 | A | C | 0.005713 | 0.521925 | 0.001008 | 1.50E-08 | 32.1 |
| rs11076320 | A | C | 0.005788 | 0.603737 | 0.001033 | 2.10E-08 | 31.4 |
| rs11124954 | G | C | -0.006 | 0.654998 | 0.001059 | 1.50E-08 | 32.1 |
| rs11984231 | A | G | -0.00702 | 0.268802 | 0.001144 | 8.40E-10 | 37.7 |
| rs12203592 | T | C | -0.0086 | 0.219648 | 0.001197 | 6.50E-13 | 51.7 |
| rs1288124 | T | C | 0.005753 | 0.513028 | 0.00104 | 3.10E-08 | 30.6 |
| rs17157586 | C | T | -0.00775 | 0.157764 | 0.00138 | 1.90E-08 | 31.6 |
| rs19573 | A | C | 0.007356 | 0.795782 | 0.001251 | 4.20E-09 | 34.5 |
| rs2079070 | G | C | -0.00655 | 0.734548 | 0.00114 | 9.10E-09 | 33.0 |
| rs2529676 | C | A | -0.00602 | 0.352311 | 0.001052 | 1.10E-08 | 32.7 |
| rs2661990 | C | A | 0.005952 | 0.36198 | 0.00105 | 1.50E-08 | 32.1 |
| rs292026 | A | G | 0.006353 | 0.270271 | 0.001134 | 2.10E-08 | 31.4 |
| rs35683183 | A | G | 0.00737 | 0.207531 | 0.001248 | 3.50E-09 | 34.9 |
| rs62523388 | A | C | 0.006862 | 0.333913 | 0.00108 | 2.10E-10 | 40.4 |
| rs6481128 | A | G | 0.005885 | 0.548275 | 0.001012 | 6.10E-09 | 33.8 |
| rs674094 | C | A | 0.007319 | 0.701296 | 0.001102 | 3.10E-11 | 44.1 |
| rs79313673 | C | T | 0.007194 | 0.209653 | 0.001243 | 7.20E-09 | 33.5 |

**B**

| SNP | Effect allele | Other allele | beta | eaf | se | p | F |
| --- | --- | --- | --- | --- | --- | --- | --- |
| rs10225528 | G | C | -0.010819 | 0.734765 | 0.00117386 | 3.10E-20 | 84.9 |
| rs13413953 | G | T | 0.00591735 | 0.359261 | 0.00108235 | 4.60E-08 | 29.9 |
| rs2120270 | G | A | -0.00628235 | 0.307991 | 0.00113798 | 3.40E-08 | 30.5 |
| rs2515938 | T | C | 0.00614417 | 0.325333 | 0.00110655 | 2.80E-08 | 30.8 |
| rs2529287 | T | C | -0.00653414 | 0.257122 | 0.00119729 | 4.80E-08 | 29.8 |
| rs3096697 | A | G | -0.00675971 | 0.233972 | 0.00122276 | 3.20E-08 | 30.6 |
| rs34713525 | A | G | -0.0060985 | 0.342223 | 0.00109454 | 2.50E-08 | 31.0 |
| rs35626515 | A | C | -0.0094116 | 0.4055 | 0.00105642 | 5.20E-19 | 79.4 |
| rs3791034 | G | A | 0.00621577 | 0.33353 | 0.00110205 | 1.70E-08 | 31.8 |
| rs4848265 | C | G | 0.00823879 | 0.176245 | 0.0013679 | 1.70E-09 | 36.3 |
| rs62084749 | T | C | 0.00630503 | 0.670273 | 0.00111098 | 1.40E-08 | 32.2 |
| rs6751655 | G | A | -0.00982071 | 0.134615 | 0.00151989 | 1.00E-10 | 41.8 |

**C**

| SNP | Effect allele | Other allele | beta | eaf | se | p | F |
| --- | --- | --- | --- | --- | --- | --- | --- |
| rs1529675 | G | A | -0.00292 | 0.407574 | 0.00051 | 9.60E-09 | 32.9 |
| rs2246873 | A | G | -0.00282 | 0.545175 | 0.000503 | 2.00E-08 | 31.5 |
| rs490972 | A | G | 0.003136 | 0.469219 | 0.000501 | 3.70E-10 | 39.3 |
| rs6461009 | C | T | 0.003631 | 0.823241 | 0.000653 | 2.70E-08 | 30.9 |
| rs6835232 | A | G | 0.002856 | 0.606683 | 0.00051 | 2.20E-08 | 31.3 |

**D**

| SNP | Effect allele | Other allele | beta | eaf | se | p | F |
| --- | --- | --- | --- | --- | --- | --- | --- |
| rs10225528 | G | C | -0.010819 | 0.734765 | 0.00117386 | 3.10E-20 | 84.9 |
| rs13413953 | G | T | 0.00591735 | 0.359261 | 0.00108235 | 4.60E-08 | 29.9 |
| rs2120270 | G | A | -0.00628235 | 0.307991 | 0.00113798 | 3.40E-08 | 30.5 |
| rs2515938 | T | C | 0.00614417 | 0.325333 | 0.00110655 | 2.80E-08 | 30.8 |
| rs2529287 | T | C | -0.00653414 | 0.257122 | 0.00119729 | 4.80E-08 | 29.8 |
| rs3096697 | A | G | -0.00675971 | 0.233972 | 0.00122276 | 3.20E-08 | 30.6 |

**E**

| SNP | Effect allele | Other allele | beta | eaf | se | p | F |
| --- | --- | --- | --- | --- | --- | --- | --- |
| rs11648192 | T | C | 0.006021 | 0.435784 | 0.000982 | 8.70E-10 | 37.6 |
| rs11896330 | A | G | -0.00594 | 0.6331 | 0.000976 | 1.10E-09 | 37.1 |
| rs12042107 | C | T | 0.005302 | 0.54923 | 0.000943 | 1.90E-08 | 31.6 |
| rs1827089 | A | G | -0.00635 | 0.778923 | 0.001132 | 2.10E-08 | 31.4 |
| rs1927902 | C | T | 0.006176 | 0.745501 | 0.001076 | 9.60E-09 | 32.9 |
| rs1941943 | T | C | 0.006312 | 0.784349 | 0.001138 | 2.90E-08 | 30.7 |
| rs2670012 | T | C | 0.005826 | 0.545137 | 0.000946 | 7.50E-10 | 37.9 |
| rs2679059 | T | A | 0.005796 | 0.595739 | 0.000958 | 1.40E-09 | 36.6 |
| rs2910829 | A | G | 0.005469 | 0.534643 | 0.000951 | 9.00E-09 | 33.0 |
| rs3026380 | C | G | 0.005715 | 0.331196 | 0.00101 | 1.50E-08 | 32.0 |
| rs34224566 | T | C | -0.00617 | 0.26614 | 0.001058 | 5.50E-09 | 34.0 |
| rs34898535 | T | C | 0.006015 | 0.377823 | 0.000966 | 4.70E-10 | 38.8 |
| rs613872 | T | G | -0.00746 | 0.825863 | 0.001235 | 1.50E-09 | 36.5 |
| rs61955196 | C | G | -0.0061 | 0.707856 | 0.001031 | 3.30E-09 | 35.0 |
| rs6739738 | T | C | -0.00563 | 0.493537 | 0.000937 | 1.90E-09 | 36.1 |
| rs7133277 | A | G | 0.005415 | 0.64071 | 0.00098 | 3.30E-08 | 30.5 |
| rs7207400 | C | T | 0.005856 | 0.271351 | 0.001052 | 2.60E-08 | 31.0 |
| rs7610856 | A | C | 0.005599 | 0.429114 | 0.000949 | 3.70E-09 | 34.8 |
| rs7617548 | C | T | -0.00643 | 0.2084 | 0.001151 | 2.30E-08 | 31.2 |
| rs968807 | G | A | -0.00527 | 0.541059 | 0.000941 | 2.10E-08 | 31.4 |

**F**

| SNP | Effect allele | Other allele | beta | eaf | se | p | F |
| --- | --- | --- | --- | --- | --- | --- | --- |
| rs10135971 | A | G | -0.00661 | 0.3386 | 0.001099 | 1.80E-09 | 36.2 |
| rs11774212 | T | C | 0.005858 | 0.515802 | 0.001043 | 1.90E-08 | 31.5 |
| rs11989077 | A | G | 0.00764 | 0.197083 | 0.001306 | 4.90E-09 | 34.2 |
| rs12507369 | C | A | 0.00616 | 0.35025 | 0.001092 | 1.70E-08 | 31.8 |
| rs12973258 | C | T | -0.00745 | 0.183691 | 0.001344 | 2.90E-08 | 30.7 |
| rs13022707 | A | C | 0.007353 | 0.23987 | 0.001219 | 1.60E-09 | 36.4 |
| rs1691471 | T | C | 0.008398 | 0.376845 | 0.001074 | 5.40E-15 | 61.1 |
| rs169413 | A | G | -0.00862 | 0.258842 | 0.001186 | 3.60E-13 | 52.9 |
| rs551243 | C | G | -0.00637 | 0.469305 | 0.001044 | 1.00E-09 | 37.3 |
| rs55773130 | C | T | 0.008482 | 0.182249 | 0.001346 | 3.00E-10 | 39.7 |
| rs62062137 | A | G | 0.006861 | 0.224664 | 0.001246 | 3.70E-08 | 30.3 |
| rs72838260 | C | T | 0.010294 | 0.094671 | 0.001773 | 6.40E-09 | 33.7 |
| rs72948529 | C | A | -0.00725 | 0.213968 | 0.001273 | 1.20E-08 | 32.4 |
| rs7615206 | C | T | -0.00752 | 0.429927 | 0.001049 | 7.70E-13 | 51.4 |
| rs9835772 | T | A | 0.007252 | 0.243634 | 0.001211 | 2.10E-09 | 35.9 |

A, The instrumental SNPs for Heavy DIY. B, The instrumental SNPs for Light DIY. C, The instrumental SNPs for none of the Heavy DIY or Light DIY. D, The instrumental SNPs for Strenuous sports. E, The instrumental SNPs for Walking for pleasure. F, The instrumental SNPs for other exercises (eg: swimming, cycling, keep fit, bowling).

**Supplementary figure 1**. MR forest plots for relationship of physical activity with epigastric pain.

A


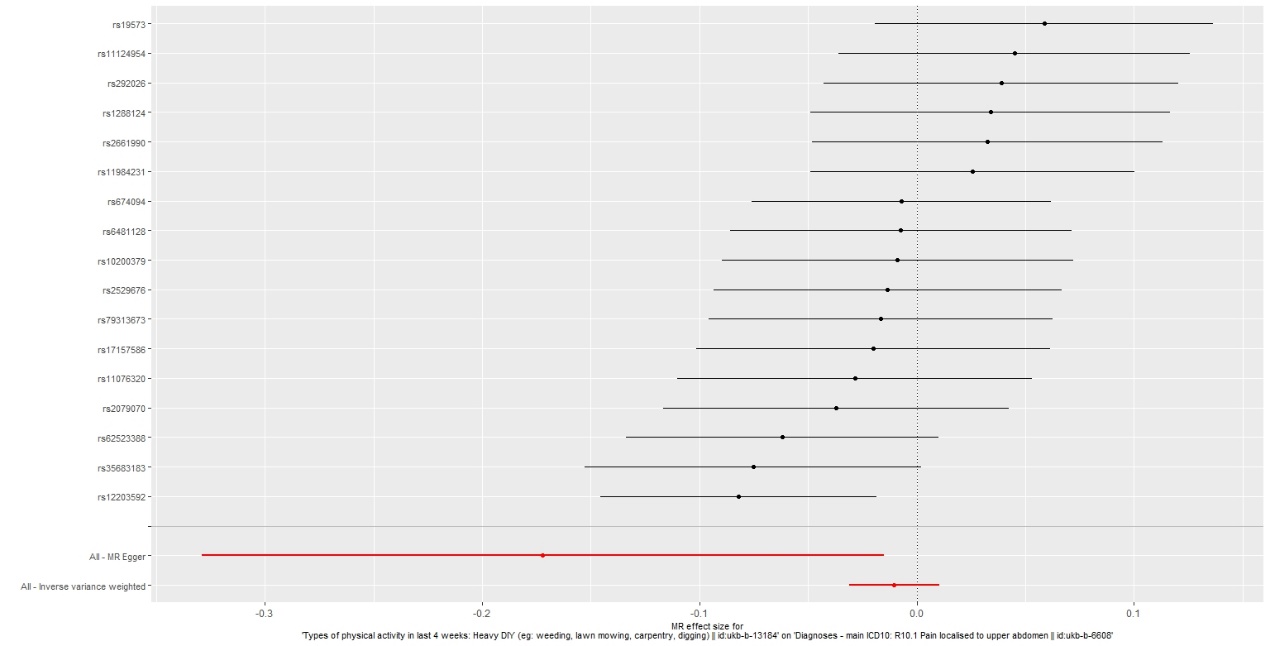


B


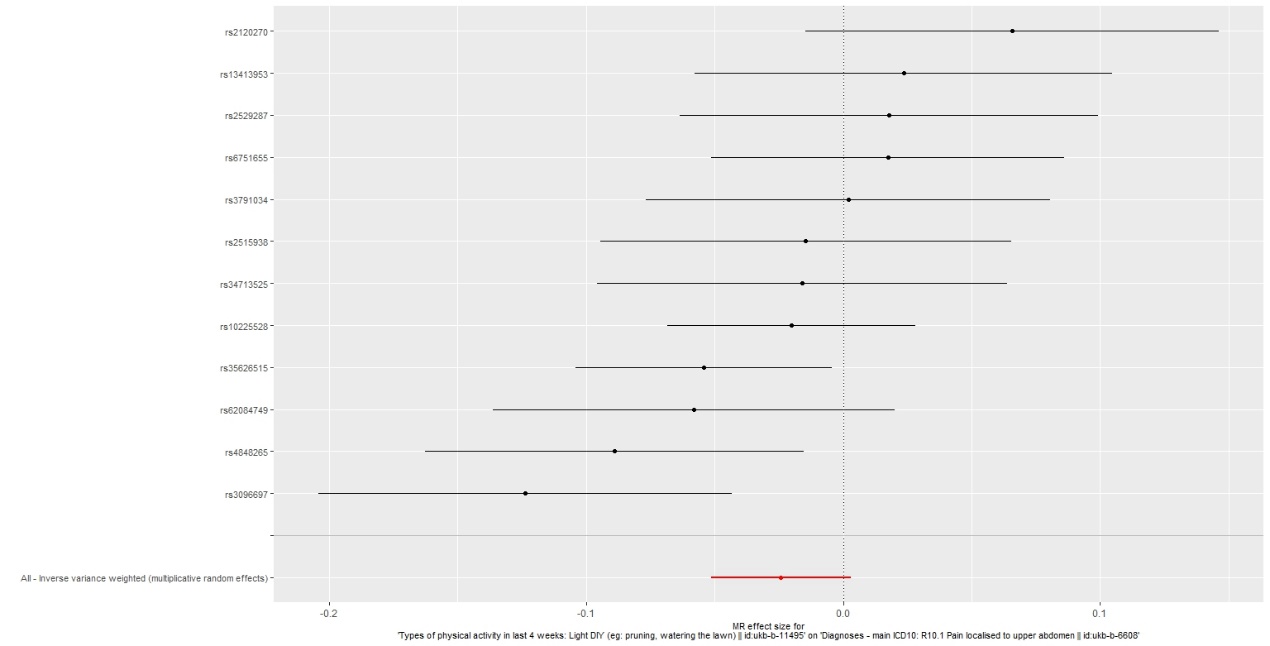


C


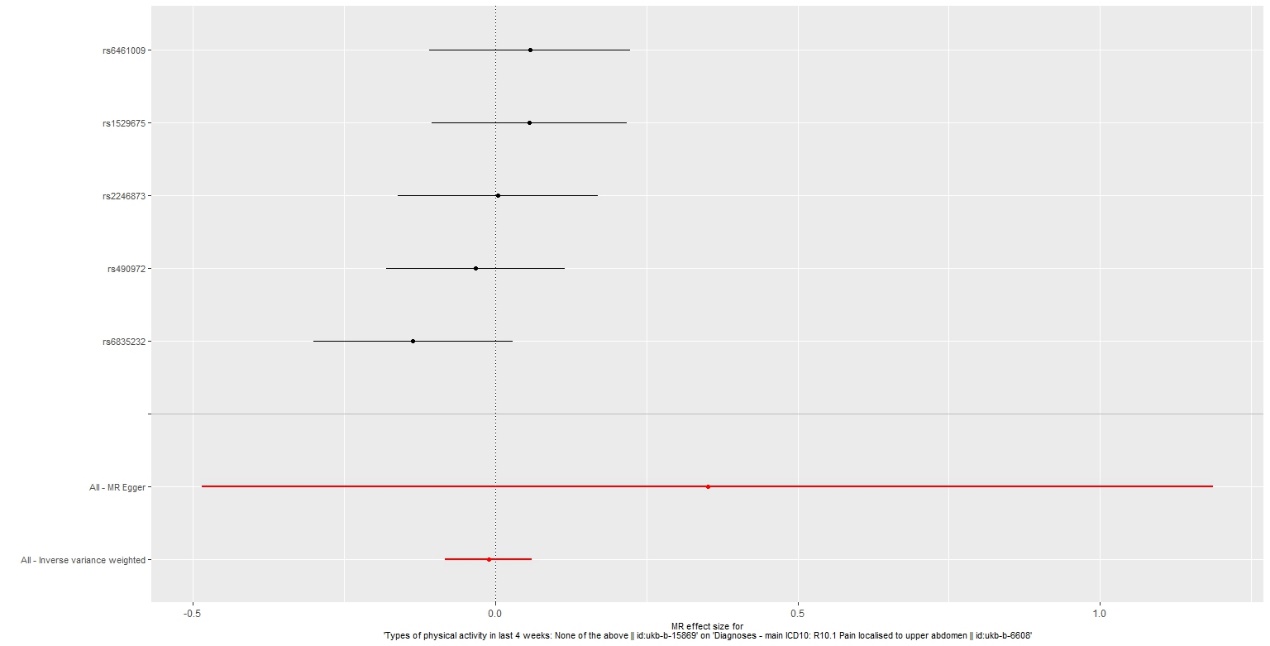


D


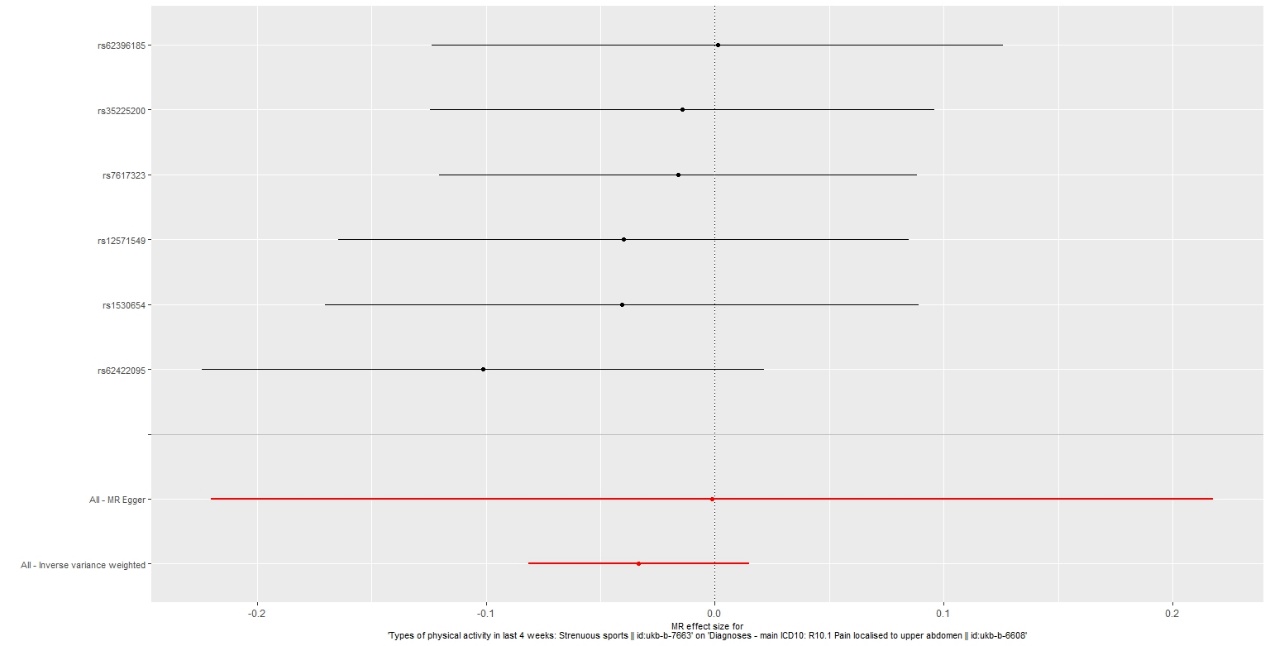


E
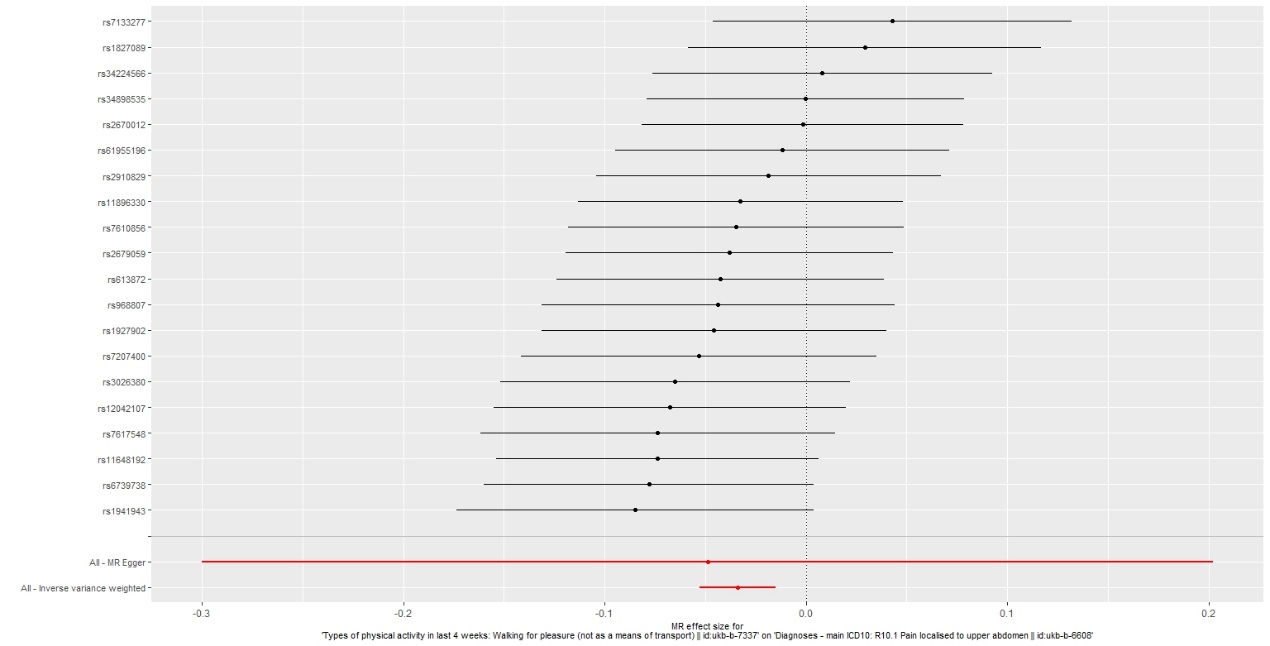


F
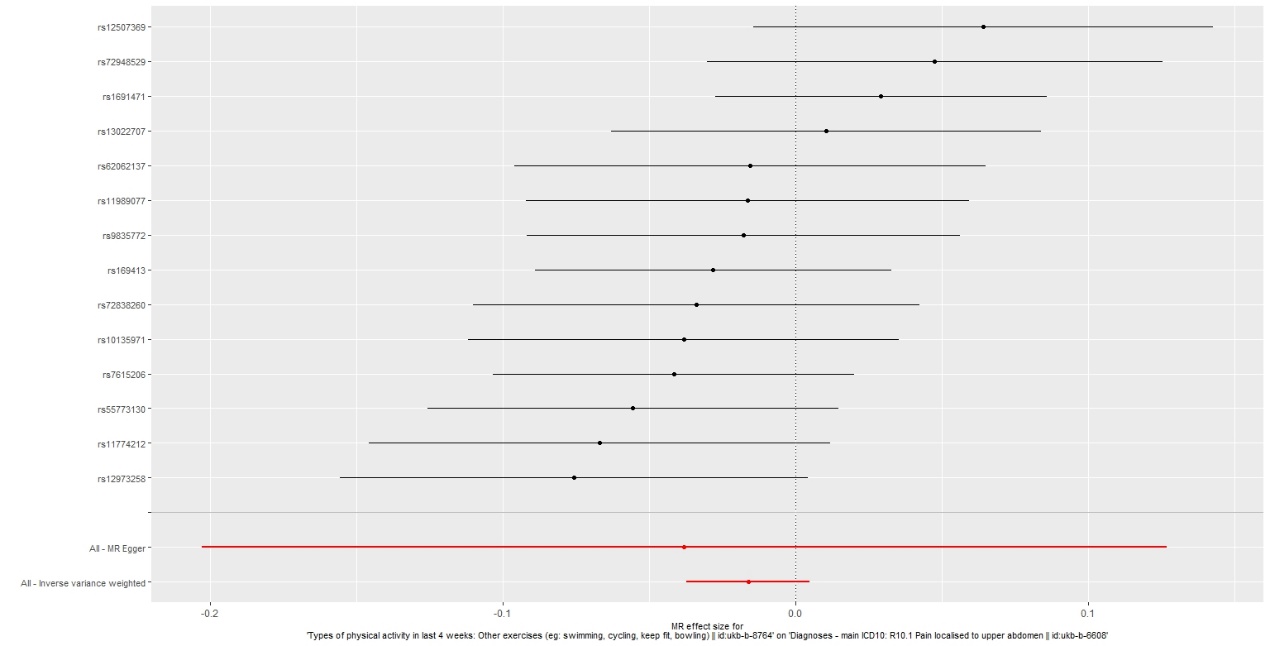


A, Forest plot of the effects of single and combined SNPs of Heavy DIY on epigastric pain. B, Forest plot of the effects of single and combined SNPs of Light DIY on epigastric pain. C, Forest plot of the effects of single and combined SNPs of none of the Heavy DIY or Light DIY on epigastric pain. D, Forest plot of the effects of single and combined SNPs of Strenuous sports on epigastric pain. E, Forest plot of the effects of single and combined SNPs of Walking for pleasure on epigastric pain. F, Forest plot of the effects of single and combined SNPs of other exercises (eg: swimming, cycling, keep fit, bowling) on epigastric pain. IVW: inverse variance weighted.

**Supplementary figure 2**. MR scatterplots for relationship of physical activity with epigastric pain.

A


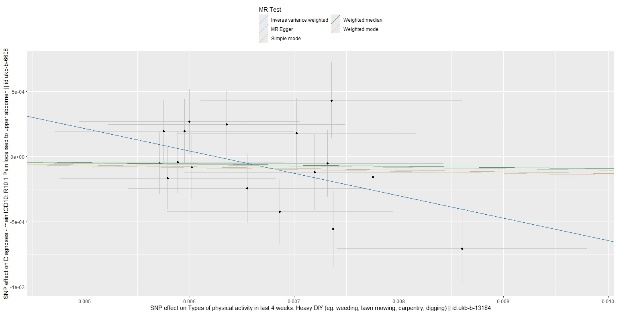


B


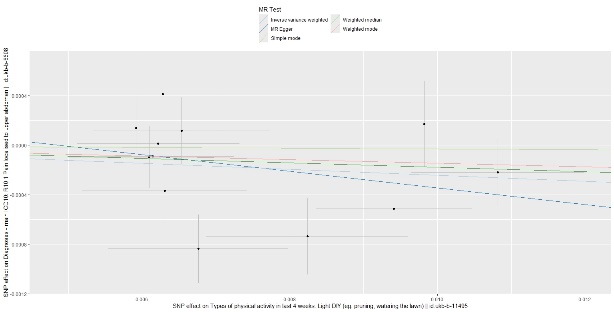


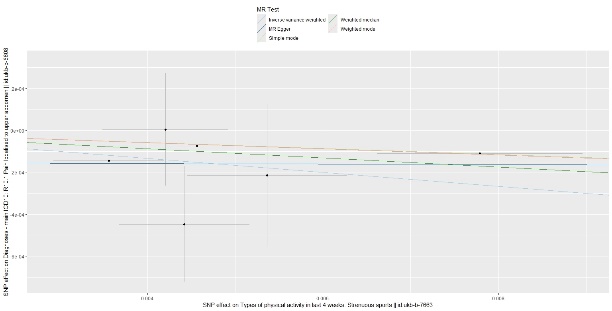
C

D


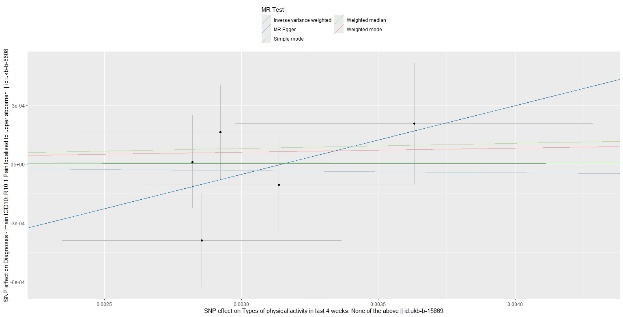


E


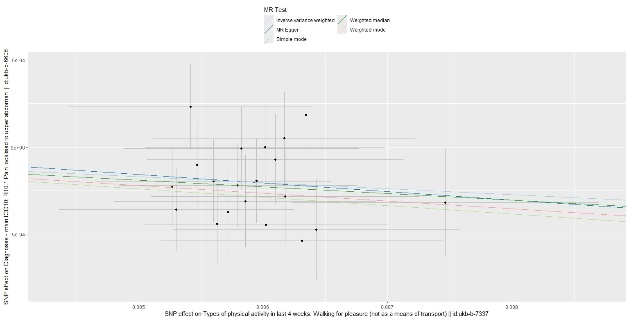


F


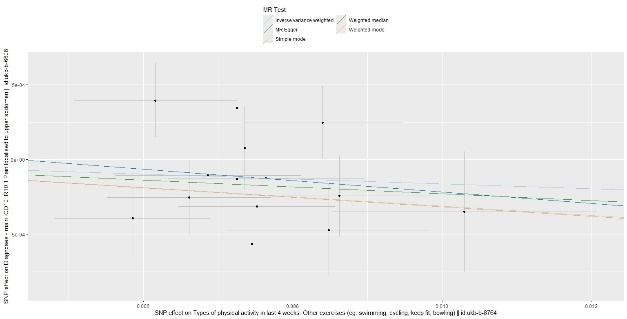


A, Scatterplot of the effects of SNPs on epigastric pain versus their effects on Heavy DIY. B, Scatterplot of the effects of SNPs on epigastric pain versus their effects on Light DIY. C, Scatterplot of the effects of SNPs on epigastric pain versus their effects on none of the Heavy DIY or Light DIY. D, Scatterplot of the effects of SNPs on epigastric pain versus their effects on Strenuous sports. E, Scatterplot of the effects of SNPs on epigastric pain versus their effects on Walking for pleasure. F, Scatterplot of the effects of SNPs on epigastric pain versus their effects on other exercises (eg: swimming, cycling, keep fit, bowling).

**Supplementary figure 3**. Leave-one-out analyses for SNPs associated with physical activity and epigastric pain.

A


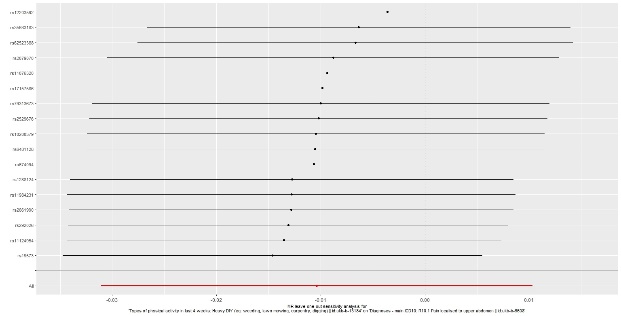


B


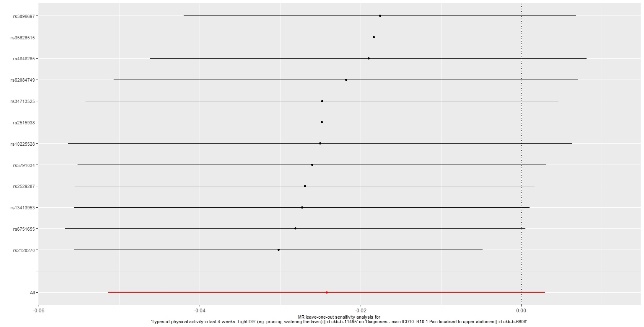


C


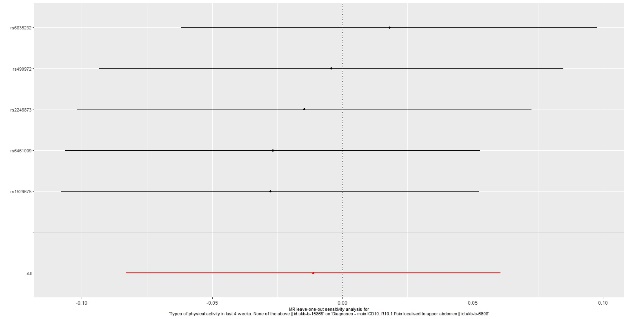


D


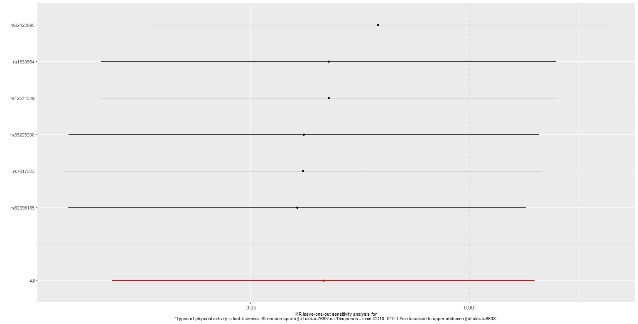


E


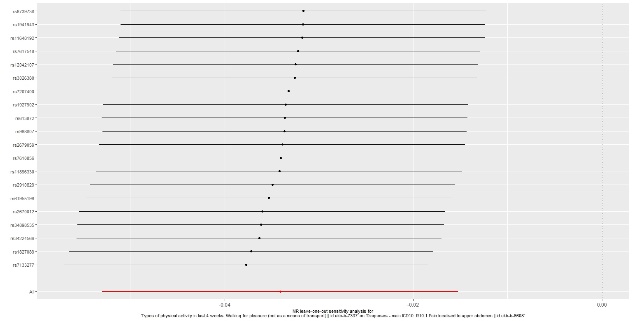


F


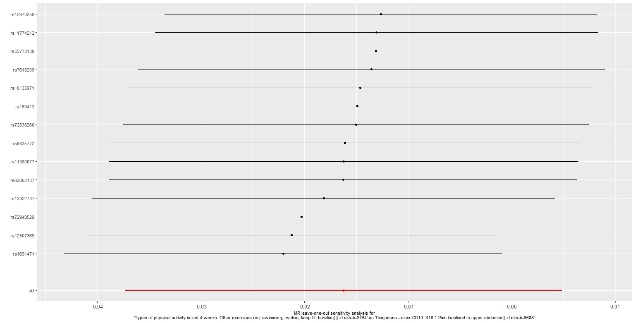


A, Leave-one-out analyses for SNPs associated with Heavy DIY and epigastric pain. B, Leave-one-out analyses for SNPs associated with Light DIY and epigastric pain. C, Leave-one-out analyses for SNPs associated with none of the Heavy DIY or Light DIY and epigastric pain. D, Leave-one-out analyses for SNPs associated with strenuous sports and epigastric pain. E, Leave-one-out analyses for SNPs associated with walking for pleasure and epigastric pain. F, Leave-one-out analyses for SNPs associated with other exercises (eg: swimming, cycling, keep fit, bowling) and epigastric pain.
